# Protocol for high-plex, whole-slide imaging of human formalin-fixed paraffin-embedded tissue using PhenoCycler-Fusion

**DOI:** 10.1016/j.xpro.2024.103226

**Published:** 2024-07-18

**Authors:** Meg L. Donovan, Niyati Jhaveri, Ning Ma, Bassem Ben Cheikh, James DeRosa, Ritu Mihani, Naomi Berrell, Jacky Y. Suen, James Monkman, John F. Fraser, Arutha Kulasinghe

**Affiliations:** 1 Queensland Spatial Biology Centre, Wesley Research Institute, Level 8 East Wing, The Wesley Hospital, Auchenflower, QLD 4066, Australia; 2 Frazer Institute, Faculty of Medicine, The University of Queensland, Brisbane, QLD 4102, Australia; 3 Akoya Biosciences, The Spatial Biology Company, Marlborough, MA, USA; 4 Critical Care Research Group, The Prince Charles Hospital, Brisbane, QLD 4032, Australia; 5 Institute for Molecular Bioscience, The University of Queensland, Brisbane, QLD 4072, Australia

**Keywords:** Single Cell, Cancer, Molecular Biology, Proteomics

## Abstract

Single-cell spatial analysis of proteins is rapidly becoming increasingly important in revealing biological insights. Here, we present a protocol for automated high-plex multi-slide immunofluorescence staining and imaging of human head and neck cancer formalin-fixed paraffin-embedded (FFPE) sections using PhenoCycler-Fusion 2.0 technology. We describe steps for preparing human head and neck cancer FFPE tissues, staining with a panel of immunophenotyping markers, and Flow Cell assembly. We then detail procedures for setting up for a PhenoCycler-Fusion run, post-run Flow Cell removal, and downstream analyses.

For complete details on the use and execution of this protocol, please refer to Jhaveri et al.^1^

## Highlights

• Protocol for preparing and staining human FFPE tissue slides for PhenoCycler-Fusion
• Flow Cell assembly and preparation for a PhenoCycler-Fusion run
• Steps for post-PhenoCycler-Fusion run Flow Cell removal for same-slide H&E staining
• Example analysis workflow for cell segmentation, phenotyping, and spatial analyses

## Abstract (editor highlights)

Publisher’s note: Undertaking any experimental protocol requires adherence to local institutional guidelines for laboratory safety and ethics.

## Graphical abstract

**Figure:**
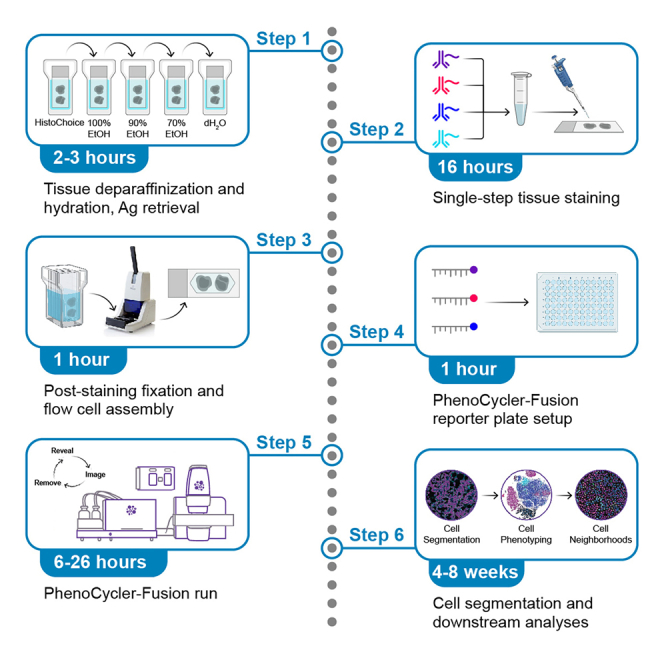

## Teaser

Single-cell spatial analysis of proteins is rapidly becoming increasingly important in revealing biological insights. Here, we present a protocol for automated high-plex multi-slide immunofluorescence staining and imaging of human head and neck cancer FFPE sections using PhenoCycler-Fusion 2.0 technology. We describe steps for preparing human head and neck cancer FFPE tissues, staining with a panel of immunophenotyping markers, and Flow Cell assembly. We then detail procedures for setting up for a PhenoCycler-Fusion run, post-run Flow Cell removal, and downstream analyses.

## Before you begin

The protocol below describes the specific steps for applying the PhenoCycler-Fusion 2.0 technology to head and neck cancer tissue samples. We have also run this protocol on healthy tonsil tissue and skin samples. A key requirement for this protocol is having the PhenoCycler-Fusion instrument as well as the PhenoCycler-Fusion associated antibodies, reagents, and accessories. Protocol steps have been partially modified from the Akoya Biosciences PhenoCycler-Fusion User Guide.^2^ PhenoCycler-Fusion is an iteration of the Co-detection by indexing (CODEX) technology.^3^ Users will have to modify and optimize the necessary antibody panels and custom markers as required for their studies.

Ensure formalin-fixed paraffin-embedded (FFPE) tissue sections are cut at a thickness of 5 μm and are positioned in the center of the charged microscope slides (as listed in the key resources table). Pre-heat oven to 60°C prior to starting the protocol.

### Institutional permissions

Permissions from the relevant institutions must be obtained before using this protocol. This study has Human Research Ethics Committee approval (UHREC #2000000494) and University of Queensland ratification.

**CRITICAL:** Ethical and institutional review board approval is required for working with human tissue specimens and relevant institutional and national regulations must be adhered to.

## Key resources table

**Table:** 

REAGENT or RESOURCE	SOURCE	IDENTIFIER
**Chemicals, peptides, and recombinant proteins**		

16% Paraformaldehyde (PFA)	ProSciTech	C004
1x PBS	Life Technologies	14190144
Methanol	Sigma-Aldrich	34860-1L-R
DMSO, ACS reagent, ≥99.9%	Sigma-Aldrich	472301-4L
Ethanol or reagent alcohol	POCD or Merck	ABSETHANOL5LTPL or 793175
HistoChoice clearing agent	VWR	H2779-1L
ddH_2_O	Fisher Scientific	10977023

**Software and algorithms**		

QuPath (v0.5.0)	Bankhead et al.^4^	https://qupath.readthedocs.io/en/stable/docs/intro/installation.html
StarDist	Schmidt et al.^5^	https://github.com/stardist/stardist
Scanpy	Wolf et al.^6^	https://scanpy.readthedocs.io/en/stable/
Rapids	Nolet et al.^7^	https://github.com/rapidsai
Scimap	Nirmal et al.^8^	https://github.com/labsyspharm/scimap
rapids_singlecell	Dicks et al.^14^	https://github.com/scverse/rapids_singlecell/tree/v0.10.4

**Other**		

PhenoCycler-Fusion (includes both the PhenoCycler-Fusion and the PhenoImager)	Akoya Biosciences	https://www.akoyabio.com/phenocycler/
Barcode-conjugated antibodies and reporters	Akoya Biosciences	https://my.akoyabio.com/ccrz__ProductList?categoryId=a2Uf4000001iVWkEAM&cartId=35f925d2-fe8c-4824-bfa4-522eaee17cbf&store=AkoyaBio&cclcl=en_US
Sample kit for PhenoCycler-Fusion (includes the hydration buffer, staining buffer, storage buffer, N blocker, J blocker, G blocker, S blocker, fixative reagent aliquots, and Flow Cells)	Akoya Biosciences	7000017
10x buffer kit for PhenoCycler-Fusion (includes the PhenoCycler-Fusion buffer and buffer additive)	Akoya Biosciences	7000019
96-well plates for PhenoCycler	Akoya Biosciences	7000006
96-well plate seals for PhenoCycler	Akoya Biosciences	7000007
Assay reagent for PhenoCycler	Akoya Biosciences	7000002
Nuclear stain for PhenoCycler	Akoya Biosciences	7000003
10x AR9 buffer or 10x AR6 buffer	Akoya Biosciences	AR9001KT or AR6001KT
Flow Cells for PhenoCycler-Fusion (2 pack or 10 pack)	Akoya Biosciences	240204 or 240205
Flow Cell Assembly Device	Akoya Biosciences	N/A
Leica Slide White Apex superior adhesive or Fisherbrand Superfrost plus glass microscope slides	Leica or Fisherbrand	3800080 or 12-550-15
Slide staining box	VWR	M918-2
Slide staining set	ProSciTech	H444-SET
Oven	Labec	https://labec.com.au/product/general-purpose-ovens-non-fan-forced-up-to-200oc300oc/
Instant Pot pressure cooker	Instant Brands	112-0181-01-AU
Coplin jars	ProSciTech	HL44208-X
LabStar mini personal centrifuge	LabGear	LABG3000001
1.5 mL Eppendorf tubes	Merck	HS4323
Opaque 1.5 mL tubes	Merck	HS4323K
Kimwipes	ProSciTech	L4104
Parafilm	Merck	P7543
Compressed air duster	Officeworks	LASCL1827F
15 mL, 50 mL conical tubes	Merck	CLS430791, CLS430829
Magnetic stir bars	ProSciTech	L34-408
Magnetic stirrer	Merck	Z671886
S1 pipet filler	Thermo Scientific	9541
5 mL, 10 mL, 25 mL serological pipettes	Merck	CLS4487, CLS4488, CLS4489
Aluminum foil	Officeworks	ICBHAF150

## Materials and equipment

**Table:** Staining Cocktail

Reagent	Storage conditions	Volume for 1x slide
Total antibody volume	N/A	Variable (1 μL of each antibody if used at recommended 1:200 dilution)
G Blocker	−20°C	5 μL
S Blocker	−20°C	5 μL
J Blocker	4°C	5 μL
N Blocker	4°C	5 μL
Staining Buffer	4°C	Variable (depends on total antibody volume)
**Total volume**	**-**	**200 μL**

Maximum time for storage: use immediately.

**Table:** Final Fixative Solution

Reagent	Storage conditions	Volume for 5x slides
1x PBS	22°C	1000 μL
Fixative Reagent	^−^20°C	20 μL

Maximum time for storage: use immediately.

**Table:** 1x PhenoCycler-Fusion Buffers

Buffer	Reagent	Storage conditions	Volume	Final volume
(A) 1x PhenoCycler-Fusion Buffer with additive	10x PCF Buffer	22°C	100 mL	1 L
	Buffer Additive	22°C	100 mL	
	ddH_2_O	22°C	800 mL	
(B) 1x PhenoCycler-Fusion Buffer (no additive)	10x PCF Buffer	22°C	100 mL	1 L
	ddH_2_O	22°C	900 mL	

Maximum time for storage: 2 weeks at 22°C.

**Table:** Reporter stock solution

Reagent	Storage conditions	1x cycle
1x PhenoCycler-Fusion Buffer (no additive)	22°C	273 μL
Assay Reagent	^−^20°C (4°C once thawed)	25 μL
Nuclear Stain	^−^−20°C (4°C once thawed)	2 μL
**Total volume**	**-**	**300 μL**

Maximum time for storage: 2 weeks at 4°C (protected from light).

## Step-by-step method details

### Tissue deparaffinization, hydration, and antigen retrieval

**Timing: 2–3 h**

This section is for the deparaffinization, hydration, and antigen retrieval of FFPE tissue slides.

1. Place the sample slide(s) in the slide staining rack.
2. Bake sample slide(s) in an incubator at 60°C for 1 h to melt paraffin.

***Note:*** To improve tissue adherence to slide, increase baking time. Different tissue types may require different baking times.

***Note:*** Organic solvents can be used for up to two weeks before they should be changed. Monitor volumes as alcohol will evaporate over time. Dispose of used solvents in dedicated waste containers following institutional protocols.

**CRITICAL:** It is highly recommended to perform this procedure under a fume hood as organic solvents are highly volatile.

**CRITICAL:** Allow the Hydration, Staining, and Storage Buffers to equilibrate to 22°C before starting the protocol. Prepare all buffers ahead of time to prevent sample degradation.

***Alternatives:*** HistoChoice Clearing Agent can be substituted with Xylene. Ethanol can be substituted with Reagent Alcohol.

3. Prepare containers (50 mL or 200 mL if using small or large Coplin jars respectively) containing the required volume of the following solvents:
  a. HistoChoice Clearing Agent (5 min).
  b. HistoChoice Clearing Agent (5 min).
  c. 100% Ethanol (5 min).
  d. 100% Ethanol (5 min).
  e. 90% (v/v) Ethanol (5 min).
  f. 70% (v/v) Ethanol (5 min).
  g. 50% (v/v) Ethanol (5 min).
  h. 30% (v/v) Ethanol (5 min).
  i. ddH_2_O (5 min).
  j. ddH_2_O (5 min).
  k. 1xAR9 (dilute stock 10xAR9 with ddH_2_O).
4. Dip the slide(s) in first solvent (listed above as Step 3a) 2x, then immerse for 5 min. Continue with steps listed above (Steps 3a-k).
5. After adding slides to 1xAR9, cover Coplin jar with foil to prevent vapor entering from the pressure cooker.
6. Fill the Instant Pot pressure cooker with ∼1 L of ddH_2_O.
7. Place the covered Coplin jar containing the slides into the pressure cooker.
8. Secure the lid of the pressure cooker and set to the high-pressure protocol and let the samples cook for 20 min.
9. After the pressure cooker incubation, **carefully** release the pressure by using the vent.

**CRITICAL:** It is highly recommended to wear face-shield and heat proof gloves while releasing the pressure.

***Alternatives:*** If a lower pH antigen retrieval buffer is required, an alternative reagent is the AR6 buffer.

***Alternatives:*** The purpose of the Instant Pot pressure cooker is to perform heat-induced antigen retrieval. Other equipment that could be used for this step includes a water bath, vegetable steamer, a rice cooker, or microwave.

10. Carefully remove the vessel from the pressure cooker and equilibrate the samples to 22°C for at least 30 min.
11. Prepare Coplin jars with the following reagents.
  a. ddH_2_O (Rinse).
  b. ddH_2_O (2 min).
  c. Hydration Buffer (2 min).
  d. Hydration Buffer (2 min).
  e. Staining Buffer (20–30 min).
12. Remove the slide(s) from the cooled 1xAR9 solution and quickly immerse it in a Coplin jar filled with ddH_2_O.
13. Place the slide(s) in a second Coplin jar filled with ddH_2_O and incubate for 2 min. Continue with steps as listed above (Steps 11a-e).

### Tissue staining

**Timing: overnight (approximately 16 h)**

This section is for the primary antibody staining of FFPE tissue slides.

**CRITICAL:** It is good practice to optimize the concentration of each antibody and validate appropriate staining prior to running full PhenoCycler-Fusion panel.

14. Prepare Staining Cocktail (briefly spin down antibody vials using a benchtop centrifuge prior to adding; 5 s at 2,350 × *g*) during the above 20–30 min incubation in Staining Buffer (Step 11e). Keep Staining Cocktail on ice until ready to use.
15. Prepare Slide Staining Box. Fill the tray with ddH_2_O until the bottom layer is covered (to a depth of 1 cm).

***Note:*** The addition of water into the Slide Staining Box will create a humidified environment, limiting evaporation.

16. Cut a rectangular piece of parafilm roughly the size and shape of the non-label portion of the sample slide.
17. Remove sample slide from the Coplin jar containing Staining Buffer and use a Kimwipe to carefully and gently absorb excess buffer.
18. Place sample slide on the tray of the Slide Staining Box.
19. Quickly dispense the 200 μL of the Antibody Cocktail to the top corner of the sample slide.

**CRITICAL:** Ensure that the liquid covers the entire tissue and that there are no bubbles.

20. Place prepared parafilm over the tissue that is covered in Antibody Cocktail.

***Note:*** The purpose of using parafilm is to ensure even staining of the tissue sample and limit evaporation.

21. Carefully place the lid on the Slide Staining Box.
22. Incubate overnight (approximately 16 h) at 4°C. Ensure box is placed on a stable surface and is not disturbed during incubation.
23. Ensure methanol (100%) is in freezer.

### Post-staining fixation and Flow Cell assembly

**Timing: 1 h**

This section is for the post-staining fixation of FFPE tissue slides and Flow Cell assembly.

24. Prepare Coplin jars with the following reagents:
  a. Staining Buffer (2 min).
  b. Staining Buffer (2 min).
  c. 1.6% PFA in Storage Buffer (10 min).
  d. 1x PBS (Rinse).
  e. 1x PBS (Rinse).
  f. 1x PBS (Rinse).
  g. Ice-cold 100% methanol on ice (5 min).
  h. 1x PBS (Rinse).
  i. 1x PBS (Rinse).
  j. 1x PBS (Rinse).

**CRITICAL:** Ensure the 1.6% PFA in Storage Buffer is made up fresh within 30 min of use.

**CRITICAL:** It is highly recommended to make the PFA dilution under a fume hood.

***Note:*** Methanol dries tissue faster than other buffers so transfer slides between solutions efficiently.

25. Gently remove the parafilm and place the sample slide(s) in the first Coplin jar containing Staining Buffer (Step 24a). Lift and immerse the sample slide(s) 2–3 times to ensure the removal of the Antibody Cocktail from both sides of the slide(s). Continue with steps as listed above (Steps 24a-j).
26. Add 1 mL of 1x PBS to Eppendorf tube for every 5 slides being prepared.
27. Retrieve one aliquot of Fixative Reagent tube from storage in −20°C freezer (one tube for every 5 samples).

***Note:*** Each Fixative Reagent tube should contain 20 μL of reagent total. Cut each tube selected for use from the tube strip.

**CRITICAL:** Do not thaw the entire strip of the Fixative Reagent tubes, just cut off the number of tubes required. Do not remove Fixative Reagent ahead of time. Let the individual 20 μL aliquot melt quickly between gloved fingers. Each tube is for single use; do not re-freeze.

28. Briefly spin down the Fixative Reagent using the benchtop centrifuge (5 s at 2,350 × *g*) to collect any liquid from the cap.
29. Dilute the 20 μL of the Fixative Reagent in 1 mL of 1x PBS to make the Final Fixative Solution.
30. Mix thoroughly or vortex the solution.
31. Cut a rectangular piece of parafilm roughly the size of the glass microscope slide.
32. Rinse and dry the Slide Staining Box if this has not already been done.
33. Remove the sample slide(s) from the Coplin jar and place each on the tray of the Slide Staining Box.

***Note:*** Use a Kimwipe to carefully and gently absorb excess buffer.

34. Add 200 μL of Final Fixative Solution to the top corner of each sample slide. Cover the entire section with reagent.

***Note:*** Do not pipette the solution directly onto the tissue and ensure there are no bubbles.

35. Carefully place the parafilm over the Final Fixative Solution on the sample slide.
36. Place lid on the Slide Staining Box and incubate for 20 min at 22°C.
37. Prepare Coplin jars with the following reagents:
  a. 1x PBS (Rinse).
  b. 1xPBS (Rinse).
  c. 1xPBS (Rinse).
  d. Storage Buffer.
  e. 1xPBS (10 min).
  f. 1x PhenoCycler Buffer with no additive (10 min).

***Note:*** Stained slides can be stored in Storage Buffer for <5 days at 4°C but must be brought to 22°C in 1x PBS before continuing with the protocol.

**Figure 1:**
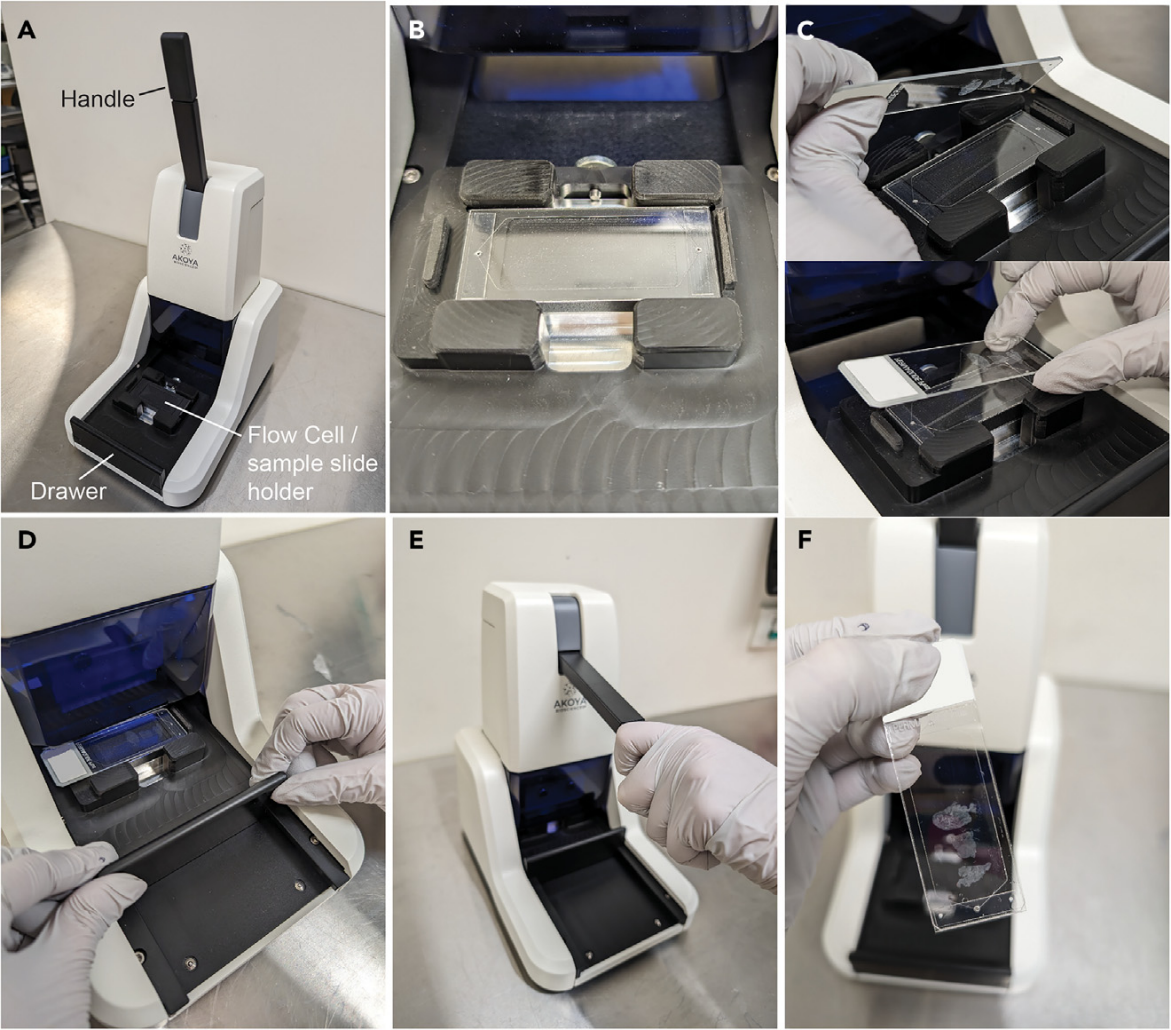
Attachment of Flow Cell to sample slide using the Flow Cell Assembly Device (A) Flow Cell Assembly Device with drawer, handle, and Flow Cell/sample slide holder indicated. (B) Placement of the Flow Cell (adhesive side facing up). (C) Placement of the sample slide (tissue side facing down) above the Flow Cell in the ‘drawer’ of the Flow Cell Assembly Device. (D) Closure of the drawer into assembly position. (E) Attachment of Flow Cell to sample slide using the Flow Cell Assembly Device. (F) Assembled Flow Cell attached to sample slide.

38. Remove the sample slide(s) from the Slide Staining Box, gently remove the parafilm, and place in the first Coplin jar containing 1x PBS (Step 37a). Lift and immerse the sample slide 2–3 times to ensure Fixative Solution is removed. Continue with steps as listed above (Steps 37a-d).
39. Attaching the Flow Cell to sample slide (refer to Figure 1).
  a. When ready to start experiment, lift and immerse the sample slide 2–3 times in PBS and incubate for 10 min (Step 37e).
  b. Remove sample slide from the PBS and carefully wipe around the sample tissue using Kimwipes. Do not leave tissue exposed to the air for extended periods.
  c. Open packet containing the Flow Cell and remove the protective plastic covering each side.
  d. Pull out the ‘drawer’ of the Flow Cell Assembly Device.
  e. Place the Flow Cell (adhesive side facing up) in the ‘drawer’ of the Flow Cell Assembly Device.
  f. Place the sample slide (tissue side facing down) on the Flow Cell. Ensure the edges of the sample slide and Flow Cell are aligned. The sample slide label should be on the left of the device.
  g. Carefully push the ‘drawer’ closed into the assembly position.
  h. Lower the Flow Cell Assembly Device handle halfway (until some resistance is felt) and pause for 30 s.
  i. Lower the Flow Cell Assembly Device handle all the way down and hold for 2 min.
  j. Raise the Flow Cell Assembly Device handle, pull out the ‘drawer’, and remove the Flow Cell that is attached to the sample slide.
  k. Inspect Flow Cell and sample slide for cracks or misalignment.
  l. Submerge the Flow Cell attached to the sample slide in the Coplin jar with 1x PhenoCycler-Fusion buffer with no additive for 10 min (Step 37f).

**CRITICAL:** Attaching the Flow Cell to the sample slide is a very important step. Failure to perform the attachment correctly can lead to a broken Flow Cell and/or broken sample slide, misaligned Flow Cell and sample slide with the Flow Cell Carrier, etc.

***Note:*** Be sure to remember which side of the Flow Cell is adhesive. Once the protective plastic is removed, hold the Flow Cell only by the edges and avoid touching the sticky adhesive.

### PhenoCycler-Fusion reporter plate setup

**Timing: 1 h**

This section is for the preparation of the PhenoCycler-Fusion reporter plate.

**CRITICAL:** Make sure that Reporters in the same cycle are paired with unique fluorescent dyes (i.e., one Reporter for each Cy5/AF647, ATTO550, and AF750 fluorophores). If two reporters are paired with the same dye in a cycle, they will be revealed at the same time in one fluorescence channel, making it impossible to distinguish the signal coming from each of the two corresponding biomarkers. Biomarkers targeting proteins that co-localize or co-express (i.e., on the same cell types or structures) should be separated into different cycles, regardless if the corresponding fluorophore is the same or different.

40. Prepare 1 L of 1x PhenoCycler Buffer with Buffer Additive. Mix by using a magnetic stir bar. This buffer will be added to the specific reagent bottle that is attached to the PhenoCycler-Fusion instrument.
41. Prepare 100 mL of 1x PhenoCycler Buffer (with no additive).

***Note:*** Do not shake to mix buffers to avoid bubble formation. If using a bottle to mix, do not invert a lid-screwed bottle to mix to avoid leakage. If any spills occur, clean with 70% ethanol.

***Note:*** Do not filter. The above 1x buffer solutions should be stored at 22°C and will be stable for 2 weeks.

42. Prepare the Reporter Stock Solution based on the total number of cycles (including 2x blank cycles) for the experiment in an amber 1.5 mL tube.
43. After adding all reagents, mix by gently inverting the Reporter Stock Solution tube a few times.

**CRITICAL:** Prevent the formation of bubbles. Do not shake or vortex the solution vigorously.

44. Prepare blank cycles. Pipette 245 μL of Reporter Stock Solution into two wells within row H (i.e., H1 and H2) as designated in the well plate layout.
45. Prepare the Reporter Master Mix for Each Cycle.
  a. For each cycle, label an opaque 1.5 mL tube with the associated cycle number or well number (for example, “A1”).
  b. Add 5 μL of the specific Reporter Stock Solution to each opaque tube depending on what is required for that cycle.
  c. The volume of Stock Solution will vary depending on whether 1 (245 μL), 2 (240 μL), or 3 (235 μL) Reporters will be revealed, however the total volume will be 250 μL.
46. Put Reporters in an ice bucket before use.
47. Briefly spin tubes down using a benchtop centrifuge (5 s at 2,350 × *g*).
48. Add 5 μL of each Reporter to its corresponding opaque tube.
49. Mix the contents of the tube by gently pipetting up and down or gently inverting the tube.

**CRITICAL:** Prevent the formation of bubbles. Do not shake or vortex the solution vigorously.

50. Once all tubes have been prepared, obtain the 96-well plate.
51. Pipette 245 μL of Reporter Master Mix from each tube into its corresponding well on the 96-well plate.

**CRITICAL:** Use caution when pipetting into the plate; do not touch, drip, or pipette into wells other than the corresponding designated one. Any cross-contamination will alter the staining profile. Do not use wells that have been contaminated. In the "PhenoCycler Experiment Designer" (PED) app, use the skip wells feature to skip the contaminated wells. Prevent the formation of bubbles while pipetting.

52. Attach the foil plate seal to the plate.
  a. Remove the adhesive layer from a foil plate seal.
  b. Cover the entire plate (or just wells containing Reporter Master Mix) and do not move or tear the foil once it has adhered to the plate.
  c. To ensure optimal sealing, carefully press down on top of each filled well.
53. Log into the computer attached to the PhenoCycler-Fusion and turn on PhenoCycler-Fusion.
54. Double-click on the “PhenoCyler Experiment Designer” (PED) app located on the PhenoCycler-Fusion Acquisition PC desktop to open the Experiment Designer.
55. To create a new experiment design, select the “New” button on the Experiment Designer main screen.
56. Select the name of an available study from the "Select or create a study for the experiment" list. If you are opening the application for the first time, this list will be blank.
57. To add a new study, enter the name in the "Create a new study field" and hit the “+” button.
58. With the appropriate study selected, enter the name of the experiment in the "Enter experiment name" field and hit “Next”.
59. On selecting “Next”, “PhenoCycler Experiment Designer” screen displays. Define the "Staining Information", "Panel Content", "Starting Well", the two blank wells, and exposure times for each channel (change DAPI channel to 3 ms) on this screen.
60. Double check the starting well and the blank cycle wells in the “Well Plate Layout” area. Select “Create” to continue or “Cancel” to return to the main screen.
61. When “Create” is selected, the cycle layout/table mode displays. Each cycle consists of detection in DAPI channel plus up to three channels. The three marker channels available depend on the filter set selected in the main window; ATTO550, Cy5, AF750 (or AF488, but this is not recommended for FFPE).
62. To edit the cycle layout, click on the well button and use the “Insert”, “Skip, “Delete” options to add, skip, or remove cycles.
63. To edit the contents within a cycle/well:
  a. Select the specific well/cycle to modify. Select “Edit”. The contents of that well display on the right side of the screen.
  b. To alter the exposure times for particular markers within a cycle, edit the exposure times for individual channels appropriately.
  c. For inventoried antibodies, use the "Marker/Barcode" dropdown box to select the marker/barcode combination to be used. If an additional item needs to be added, select “Add additional item” from the dropdown box and specify the details for that item.
  d. Repeat steps 63a-c as needed to edit the layout of the wells.
  e. When finished, **select “Update” to save** **the** **layout** or select "Cancel".

***Note:*** The default exposure time is 150 ms, which is a good starting point for optimization.

### Launching the PhenoCycler-Fusion run

**Timing: 1 h set up (approximately6–26 h run time depending on number of markers, number ofPhenoCycler-Fusioncycles, tissue size, etc.)**

This section is for launching the PhenoCycler-Fusion run of stained FFPE tissue slides.

64. Open the “PIF” icon on the PhenoCycler-Fusion Acquisition PC desktop.
65. Go to “PhenoCycler-Fusion” / “Run Experiment”.
66. Link experiment layout created with “PED”.
67. Follow all prompts given by the software **carefully.**
68. When attaching reporter plate, ensure A1 is positioned at the front-left of machine.
69. When prompted to insert sample slide, carefully wipe assembled Flow Cell and sample slide with 70% ethanol and then ddH_2_O using Kimwipes prior to placing in the carrier.

**CRITICAL:** Ensure no fibers or dust is present on the surface of the assembled Flow Cell and sample slide.

70. Select “Run” and **carefully** follow all prompts given.

### Optional: Removal of Flow Cell for same slide H&E staining

**Timing: overnight (approximately 16 h) + 30 min**

This section is for removing the Flow Cell after running the PhenoCycler-Fusion for same slide H&E staining.

71. Place the tissue sample slides with the Flow Cell in a Coplin jar containing HistoChoice Clearing Agent and incubate overnight (approximately 16 h) at 22°C.
72. Separate the Flow Cell from the sample slide.
73. Prepare Coplin jars with the following reagents:
  a. ddH_2_O (rinse).
  b. ddH_2_O (rinse).
  c. ddH_2_O (rinse).
  d. ddH_2_O (rinse).
  e. ddH_2_O (rinse).
  f. 1x PhenoCycler Buffer without additive (10 min).
  g. 1x PhenoCycler Buffer without additive (10 min).
  h. 1x PhenoCycler Buffer without additive (10 min).
  i. PhenoCycler Storage Buffer (if slide not used immediately).
74. Place the sample slide in the first Coplin jar containing ddH_2_O (Step 73a). Lift and immerse the sample slide 2–3 times to ensure the removal of the HistoChoice Clearing Agent from both sides of the slide. Continue with steps as listed above (Steps 73a-i).
75. Proceed with H&E staining protocol.
76. Image on PhenoImager.

## Expected outcomes

At the completion of a PCF run, a composite qptiff file is generated with multiplex spatial data from all markers in the high plex panel at single-cell resolution. The file is best viewed using open-source whole-slide analysis software such as QuPath,^4^ which is recommended by the manufacturer due to the specific file type. Within QuPath^4^ each marker can be viewed individually or together with other markers in the panel to determine the expression pattern of the protein across the entire tissue section. Nuclear or membrane localization of the protein can be analyzed at the single-cell level as well as co-expression of proteins.

**Figure 2:**
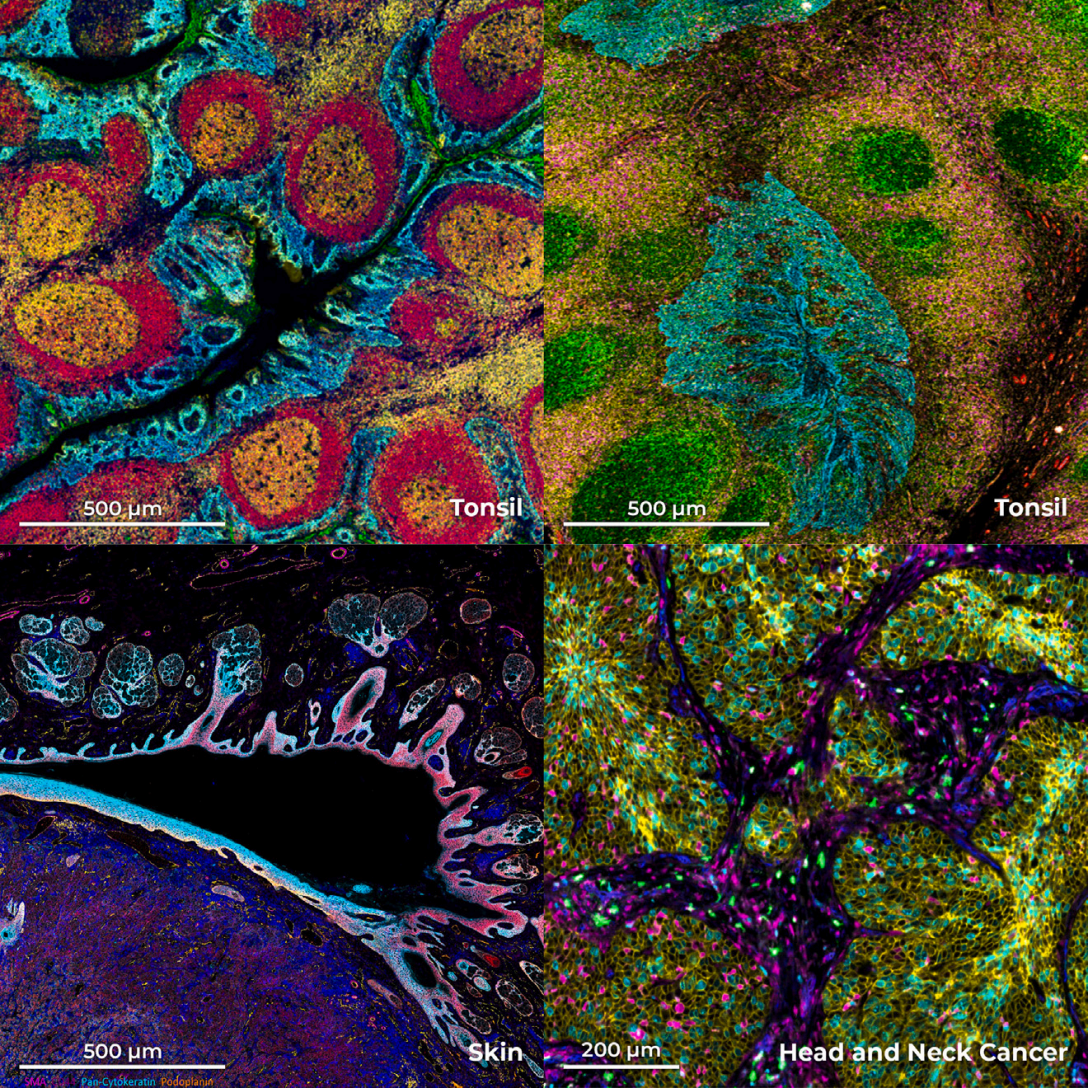
PhenoCycler-Fusion image output examples Each image depicts a tissue stained with a specific PhenoCode Discovery Panel. Upper left; Tonsil stained with the Immune Activation and Proliferation Module. Botton left; Skin stained with the Tissue Architecture Module. Upper right; Tonsil stained with the Lymphocyte Profiling Module. Bottom right; Head and neck cancer stained with the Immune Activation and Proliferation Module. Imaged at 20x resolution (0.5 microns/pixel). Scale bars are either 500 μm or 200 μm as indicated,

Panels can be constructed using predesigned panels known as PhenoCode Discovery panels (see Figure 2), designed to expedite investigations into fundamental biological inquiries. These panels encompass a range of modules tailored to specific aspects of immune profiling, lymphocyte characterization, tissue architecture elucidation, and immune activation and proliferation assessment. The panels include the Immune Profiling Core (CD4, CD68, CD20, CD11c, CD8, CD3e, CD44, CD45, HLA-A, HLA-DR, CD45RO, CD14, CD56, Ki67, PanCK), the Lymphocyte Profiling Module (T-bet, FOXP3, CD39, CD21, TOX, Granzyme B, CD107a, CD79a, TCF-1, CD38), the Tissue Architecture Module (E-cadherin, Podoplanin, Collagen IV, b-catenin1, Vimentin, Beta-actin, Caveolin, SMA, CD34, CD31), and the Immune Activation and Proliferation Module (PD-1, PD-L1, ICOS, PCNA, LAG3, IDO1, CD40, VISTA, HLA-E, IFNG) with future panels in development.

**Figure 3:**
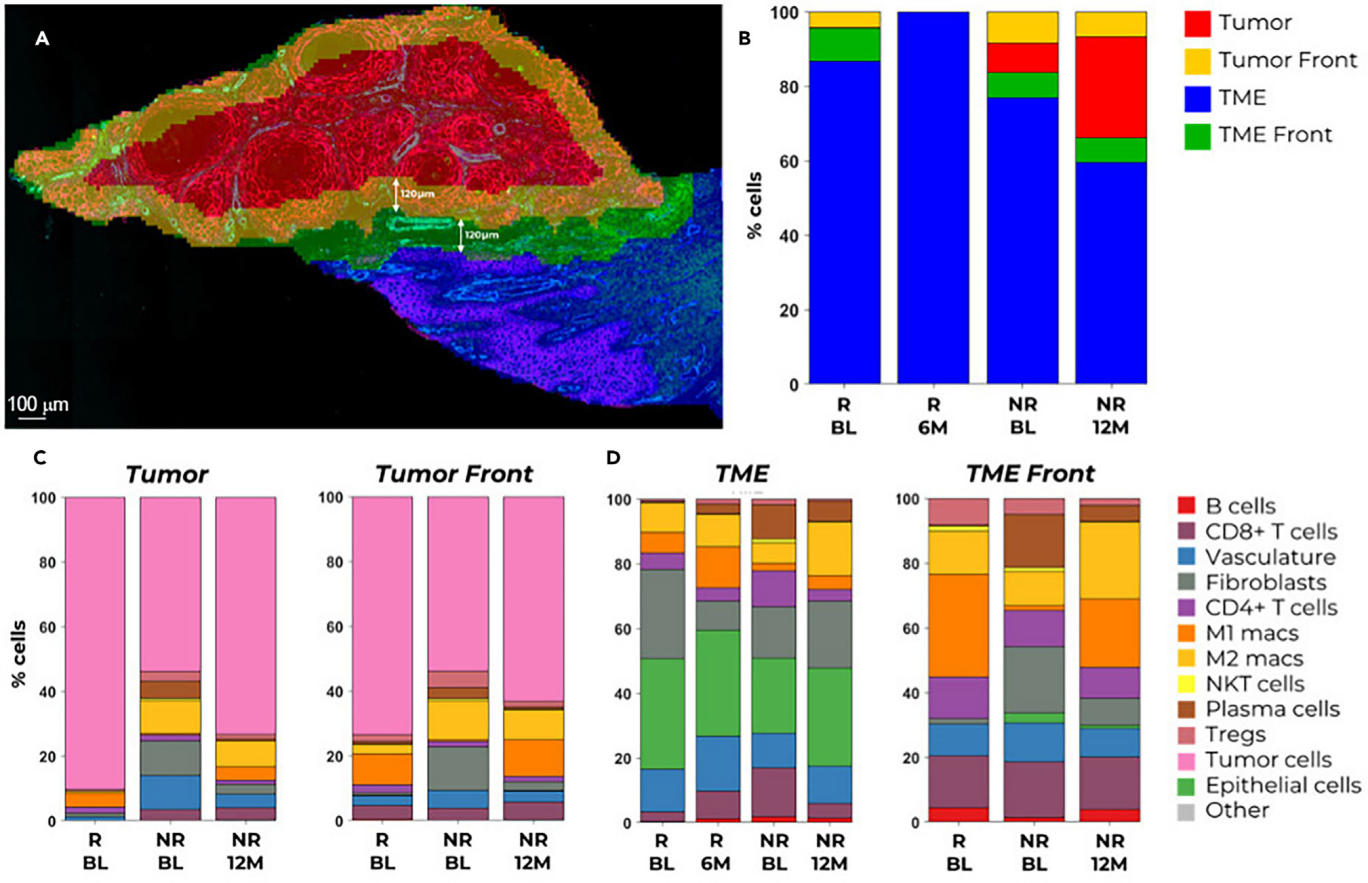
PhenoCycler-Fusion data analysis example In this preliminary analysis, two skin cancer patients were subjected to profiling both prior to and following immunotherapy, with evaluations conducted at 6–12 months post-treatment. The patient biopsy sections were segmented into tumor, tumor front, tumor microenvironment (TME) and TME front (A) and the total number of cells in each region were plotted (B). Utilizing the 60-plex panel, notable regional heterogeneity in cellular composition within the tumor and tumor front (C), as well as the TME and TME front (D) was observed. Particularly discernible were disparities in the distribution of M1 macrophages, M2 macrophages, and plasma cells between baseline (BL) and 6–12 months post-treatment across cSCC samples, distinguishing the responder (R) from the non-responder (NR). BL = baseline; R = responder; NR = non-responder. Imaged at 20x resolution (0.5 microns/pixel). The total number of cells used for analysis was ∼380,000 cells. Scale bar is 100 μm,

Each module comprises a selection of markers strategically chosen to address distinct biological questions. Additionally, the modularity of these panels permits customization through the inclusion of supplementary custom markers, ensuring adaptability to diverse experimental requirements. These panels have been extensively applied across various spatial studies, demonstrating particular efficacy in elucidating the spatio-temporal dynamics inherent in the progression of cutaneous squamous cell carcinoma (cSCC) and the subsequent response to immunotherapeutic interventions. Specifically, the aforementioned panels were integrated to discern the spatio-temporal alterations within the cSCC microenvironment (Figure 3).

## Quantification and statistical analysis

**Timing: 4–8 weeks**

This section is for downstream image quantification and statistical analysis using open-source software and packages that are readily available online. Below is one example of an analysis workflow that identifies different cell types and characterizes their spatial organization in the tissue, and it consists of four main steps: quality control, cell segmentation, cell phenotyping, and spatial analysis.

### Quality control

Before initiating data analysis, each individual image undergoes quality control through visual assessment across the whole slide using QuPath.^4^ The qualitative evaluation of each marker is conducted by comparing the signal intensity to the background and examining the specificity of the staining. Other artifacts such as out-of-focus regions, tissue folding, bubbles, or debris are manually annotated and excluded from the analysis. Additional factors that may be considered during this quality control step are outlined elsewhere.^9^

### Segmentation

The next step in image analysis is cell segmentation, which identifies individual cells in the tissue by determining their surfaces and centroid locations. Nuclear segmentation is performed in QuPath^4^ using the deep learning method StarDist^5^ on the DAPI image, employing its default parameters, and the 2D_dsb2018 model. This segmentation method can be trained on a variety of cell types, depending on the tissue of interest. To match the training set’s image resolution and improve image contrast for prediction, preprocessing steps are taken, including reducing the image size by 50% and applying Contrast Limited Adaptive Histogram Equalization (CLAHE). Cytoplasmic segmentation is achieved by expanding the nuclei with a morphological dilation of 6 μm applied to the labeled nuclear mask. The centroid of each cell is then determined using the x-y coordinates of the nuclear object’s centroid within the image. A comprehensive qualitative assessment of the segmentation is conducted for each individual slide, consistently yielding satisfactory results, which confirms the robustness and reliability of the segmentation process.^1^ Following cell segmentation, the spatial coordinates and average intensity of each marker is calculated for each cell using the segmentation masks and the marker images in QuPath^4^ and can be exported as a .csv file (i.e., the count table). When the protein is localized in the nucleus, such as Ki-67, PCNA, or FOXP3, the average intensity is derived from the nuclear mask. Conversely, for proteins localized in the cell cytoplasm/membrane, like CD4, CD3, or E-cadherin, the average intensity is calculated from the cytoplasm mask. The count table can be converted into an expression matrix, conveniently containerized within the AnnData^6^ format, commonly used for downstream single-cell spatial analyses. The expression of each protein is then z-scored across all cells in the slide, ensuring that each protein has a mean of zero and a standard deviation of one.^10^ For cell phenotyping, a specific subset of markers is usually selected based on the research question being addressed in the study.^1^^,^^9^

### Phenotyping

The conversion of multiplexed images into expression matrices draws convenient analogs to single-cell gene expression data, where protein marker intensities become analogous to gene counts. With this, well established analysis toolkits designed for single-cell gene expression data can be applied. Scanpy,^6^ can be used to phenotype cells using a workflow which combines unsupervised clustering methods with biologist-informed annotations. In this workflow, dimensionality reduction is first performed on the expression matrix using principal components analysis (PCA). The similarity of protein expression patterns between cells are then represented by constructing a k-nearest neighbors (KNN) graph using distances in the PCA embedding space. Finally, unsupervised graph-based clustering (or community detection) algorithms such as the Leiden algorithm,^11^ are used to cluster cells at a given r or resolution parameter. These are the two main parameters that can be used to determine the most suitable number of clusters for manual cluster annotation. The k parameter sets how many adjacent cells in the PCA embedding space should be considered when constructing the KNN graph. The r parameter sets how granular the clusters are, with higher r values resulting in more granular clusters. The decision for the values of k and r typically hinges on finding a sufficient number of clusters which effectively segregate cells into smaller subsets based on the differentiation of protein expression variability over technical variability. Subsequently, cell phenotypes are identified and assigned based on their distinct protein expression patterns, as revealed through a hierarchical clustering heatmap. Clusters exhibiting similar expression profiles are then combined into a single phenotype, followed by the creation of a new phenotyping heatmap.

For the purpose of visualizing more complex relationships between cells, non-linear dimensionality reduction techniques in addition to PCA are actively employed, such as uniform manifold approximation and projection (UMAP)^12^ and t-stochastic neighbors embedding (t-SNE),^13^ also conveniently implemented in Scanpy.^6^ The parameters for constructing UMAP embeddings are usually set as follows: 20 neighbors, a minimum distance of 0.05, and a spread of 0.5. Conversely, the t-SNE method is adjusted with a perplexity setting of 30, an early exaggeration of 12, and a learning rate of 200. These settings facilitate an effective reduction in the complexity of the data, allowing for the visualization of clusters of phenotypes related in high-dimensional space, on a lower two-dimensional plot.

The scalability of these analyses is an important consideration, where for a large dataset comprising of millions of cells, some of the algorithms introduced may have unfeasible runtimes. Conveniently, these algorithms can be accelerated with the use of graphics processing units (GPUs), utilizing GPU-accelerated versions, available as lower level code^7^ or through a toolkit called rapids_singlecell.^14^

### Spatial analysis

#### Cellular neighborhood

The current analysis involves quantifying the spatial interactions between various cell phenotypes through the Cellular Neighborhood method, as described in Schürch et al. (2020).^15^ This approach is pivotal in unveiling the relative distribution of different cell types within the immediate vicinity of any given cell. In this workflow, the initial step involves defining a cell’s neighborhood, specifically as its 10 nearest neighboring cells.^9^^,^^15^ Following this, for every individual cell, we compute the proportion of each cell type present within its neighborhood. This calculation results in the formation of a neighborhood matrix, wherein each cell is distinctly characterized by the cell type percentages in its immediate surroundings. Subsequently, K-means clustering is applied to all cells, utilizing these percentage-based characterizations. The final step involves computing and visually representing the average neighborhood percentages for each cellular neighborhood on a heatmap. This approach offers a deeper and more intricate insight into the spatial relationships and interactions among various cell phenotypes.

#### Proximity analysis

Another method for assessing the spatial relationships between cells involves spatial proximity analysis. This technique focuses on measuring the pairwise distances between different cell phenotypes. The spatial_distance function in Scimap^8^ effectively maps the positions of cell phenotypes relative to one another by calculating the average distance from the nearest cells of type “B” to a target cell of type “A”. Specifically, for each cell of type “A”, the distances to cells of cell type “B” are identified, and the average shortest distance to the type “A” cell is computed and attributed as a key feature of cell “A”.^8^^,^^9^ Following this, average values of this characteristic are determined for all cells of type “A” and stored in the adata.uns and used to characterize the sample or the Region Of Interest (ROI), enabling comparisons between various conditions, such as treated versus control groups.

## Limitations

The major limitation for this protocol is that it requires a PhenoCycler-Fusion system. The protocol would have to be modified for a different system with similar capabilities. Other possible limitations of the protocol include the currently limited range of PhenoCycler-Fusion barcoded antibodies. Custom conjugation of antibodies to oligonucleotide barcodes is possible but requires testing and validation.^3^^,^^10^^,^^16^ The protocol may be unsuccessful in situations where the tissue section has not adhered well to slides. Any dust or fibers caught between the Flow Cell and the tissue section may impact the image acquisition and introduce artifacts into the final qptiff image. Slide-to-slide variation in tissue section preparation and staining may also introduce challenges for downstream data analyses.

Another limitation of this protocol is that there is currently no standardized data analysis pipeline. As it stands currently, the downstream analysis of high-dimensional proteomic data is computationally intensive. High-plex data over a few markers becomes rapidly complex to analyze due to co-expression of multiple markers per cell across millions of individual cells arranged throughout the tissue samples. Analysis of the data and accurate cell typing requires bespoke analytical pipelines, as is the case for Imaging Mass Cytometry (IMC). Indeed, even single-cell RNA sequencing or spatial transcriptomics has pitfalls with reliance on reference data or manual annotation. Multiple approaches have been developed (e.g., Scimap,^8^ CSPOT,^17^ X-shift,^18^ Phenograph,^19^ MCMICRO^20^) and methods that include spatial embeddings (e.g., CELESTA,^21^ BANKSY^22^), however they all require a convergence of expert biological annotation and bioinformatics. It is an ongoing effort, and as such, it will take some time for the field to reach a consensus on a single analysis pipeline. Here, we have presented a basic method for phenotyping by clustering, but this is a rapidly expanding field, and beyond the scope of this multiplex staining protocol.

## Troubleshooting

### Problem 1

Bubbles may form on the tissue when dispensing the Antibody Cocktail onto the sample slide (Step 19).

### Potential solution

To minimize the risk of bubbles, be careful not to pipette the solution directly onto the tissue.

### Problem 2

Non-uniform staining of tissues (Step 19).

### Potential solution

Ensure the Antibody Cocktail is well mixed prior to staining sample. When adding the Antibody Cocktail to the sample slide, make sure that the liquid covers the entire piece of tissue and that there are no bubbles.

### Problem 3

Dust may get trapped on the sample slide between the tissue and the Flow Cell (Steps 39e, f).

### Potential solution

Use compressed air (e.g., ‘Compressed Air Duster’ that is used for cleaning keyboards) to remove any dust particles on the Flow Cell before attaching to the sample slide. When cleaning the sample slide (with Flow Cell attached) prior to loading into the Flow Cell Carrier (Step 70), avoid wiping directly over the holes on either side of the Flow Cell.

### Problem 4

Flow Cell may break when attaching to the sample slide (Step 39k).

### Potential solution

Check that the Flow Cell is positioned centrally in the Flow Cell Assembly Device and that the sample slide aligns correctly. Stain two serial sections of the same sample at once in case the Flow Cell breaks during the attachment step. Then the back-up sample slide can be used.

### Problem 5

Tissue may detach from the slide during the sample preparation (Steps 2–38).

### Potential solution

The baking time (Step 2) may need to be increased to allow better adherence of tissue to the charged microscope slide. Different tissue types may require different baking times. A good starting point for troubleshooting this step is to check the literature for other immunohistochemistry or immunofluorescence studies that use the same tissue type. For example, Schürch et al. (2020) specified a 1 h baking time at 70°C for human colorectal tissue sections,^15^ while Bandyopadhyay et al. (2024) utilized a 3 h baking time at 65°C for human bone marrow biopsy sections.^23^

### Problem 6

Presence of bubbles in the Flow Cell attached to sample slide after priming (Step 70).

### Potential solution

The priming step can be repeated multiple times to remove bubbles. If this does not work, the sample slide can be removed, placed in 1x PCF Buffer, and the PhenoCycler-Fusion set-up process repeated (Steps 64–70).

## Resource availability

### Lead contact

Further information and requests for resources and reagents should be directed to and will be fulfilled by the lead contact, Dr Arutha Kulasinghe (arutha.kulasinghe@uq.edu.au).

### Technical contact

Technical questions on executing this protocol should be directed to and will be answered by the technical contacts, Dr Meg Donovan (meg.donovan@wesleyresearch.org.au) or Dr Niyati Jhaveri (njhaveri@akoyabio.com).

### Materials availability

This study did not generate new unique reagents.

### Data and code availability

This study did not generate new unique datasets or code.
